# Transcranial Direct Current Stimulation (tDCS) Paired with Occupation-Centered Bimanual Training in Children with Unilateral Cerebral Palsy: A Preliminary Study

**DOI:** 10.1155/2018/9610812

**Published:** 2018-11-05

**Authors:** Tonya L. Rich, Samuel Nemanich, Mo Chen, Kathleen Friel, Timothy Feyma, Linda Krach, Tanjila Nawshin, Gregg Meekins, Bernadette T. Gillick

**Affiliations:** ^1^Department of Rehabilitation Medicine, Program in Rehabilitation Science, University of Minnesota, Minneapolis, MN 55455, USA; ^2^Department Rehabilitation Medicine, Program in Physical Therapy, University of Minnesota, Minneapolis, MN 55455, USA; ^3^Non-Invasive Neuromodulation Laboratory, MnDRIVE Initiative, University of Minnesota, Minneapolis, MN 55455, USA; ^4^Department of Psychiatry, University of Minnesota, Minneapolis, MN 55455, USA; ^5^Burke Neurological Institute, White Plains, NY 10605, USA; ^6^Weill Cornell Medicine, New York, NY 10065, USA; ^7^Blythedale Children's Hospital, Valhalla, NY 10595, USA; ^8^Gillette Children's Specialty Healthcare, St. Paul, MN 55101, USA; ^9^Courage Kenny Rehabilitation Institute-Minneapolis, Minneapolis, MN 55407, USA; ^10^Department of Neurology, University of Minnesota, Minneapolis, MN 55455, USA

## Abstract

**Objective:**

We investigated the preliminary efficacy of cathodal transcranial direct current stimulation (tDCS) combined with bimanual training in children and young adults with unilateral cerebral palsy based on the principle of exaggerated interhemispheric inhibition (IHI).

**Methods:**

Eight participants with corticospinal tract (CST) connectivity from the lesioned hemisphere participated in an open-label study of 10 sessions of cathodal tDCS to the nonlesioned hemisphere (20 minutes) concurrently with bimanual, goal-directed training (120 minutes). We measured the frequency of adverse events and intervention efficacy with performance (bimanual—Assisting Hand Assessment (AHA)—and unimanual—Box and Blocks), self-report (Canadian Occupational Performance Measure (COPM), ABILHAND), and neurophysiologic (motor-evoked potential amplitude, cortical silent period (CSP) duration, and motor mapping) assessments.

**Results:**

All participants completed the study with no serious adverse events. Three of 8 participants showed gains on the AHA, and 4 of 8 participants showed gains in Box and Blocks (more affected hand). Nonlesioned CSP duration decreased in 6 of 6 participants with analyzable data. Cortical representation of the first dorsal interosseous expanded in the nonlesioned hemisphere in 4 of 6 participants and decreased in the lesioned hemisphere in 3 of 4 participants with analyzable data.

**Conclusions:**

While goal achievement was observed, objective measures of hand function showed inconsistent gains. Neurophysiologic data suggests nonlinear responses to cathodal stimulation of the nonlesioned hemisphere. Future studies examining the contributions of activity-dependent competition and cortical excitability imbalances are indicated.

## 1. Introduction

Children with unilateral cerebral palsy (UCP) due to perinatal stroke or periventricular leukomalacia exhibit great variability in clinical presentation. This heterogeneity may be partially attributed to neuroplastic influences, both developmental and maladaptive, on the corticospinal tract (CST). Developmentally, the CST is established through competitive withdrawal of bilateral CST projection fibers early in infancy driven in part by activity-dependent influences [1]. In children with UCP, bilateral CST projection fibers do not withdraw, as would be observed in children with typical development [2]. A lack of competitive withdrawal is compounded by decreased activity of the weaker, or more affected, hand during early development [3].

In addition to activity-dependent influences on the CST during development, a potential maladaptive influence is an imbalance in interhemispheric inhibition (IHI) observed in adults with stroke, which may limit motor recovery [4]. Similarly, for some children with UCP, greater inhibition from the nonlesioned hemisphere is observed as compared to the lesioned hemisphere [5, 6]. Interventions targeting inhibition of the nonlesioned hemisphere have resulted in improvements in hand function and goal attainment [7–9]. One factor that may influence the response to novel intervention, such as combined noninvasive brain stimulation (NIBS) and rehabilitation protocols, is altered patterns of underlying brain circuitry of the CST in pediatric populations with neurologic deficits [10].

Assessments of cortical excitability using NIBS, such as transcranial magnetic stimulation (TMS), can be used to examine CST circuitry, a key biomarker in children with UCP related to hand function [11, 12]. Depending on responses obtained from each brain hemisphere, CST circuitry patterns can be described as contralateral, bilateral, and ipsilateral. Contralateral CST circuitry describes when a motor-evoked potential (MEP) is elicited from a muscle contralateral to stimulation (e.g., stimulation to the lesioned hemisphere elicits a MEP from the more affected hand and stimulation to the nonlesioned hemisphere elicits a MEP from the less affected hand) [11, 12]. Bilateral CST circuitry describes responses following stimulation to (1) the lesioned hemisphere with a MEP elicited from the more affected hand and (2) the nonlesioned hemisphere with a MEP elicited from both hands. Ipsilateral circuitry describes when children display a MEP from both hands when stimulating the nonlesioned hemisphere and no MEP from the more affected hand following stimulation to the lesioned hemisphere. Children on the contralateral CST circuitry continuum (e.g., contralateral and bilateral CST circuitry) show greater baseline function of the more affected hand [13]. However, both children with contralateral and ipsilateral circuitry patterns respond to upper limb rehabilitation [5, 8, 14, 15].

Bimanual training is one type of upper limb rehabilitation intervention used for children with all types of CST circuitry [16]. This form of rehabilitation is designed to activate the more affected (e.g., weaker) and less affected (e.g., stronger) limbs during daily living skills and goal-directed training [17]. Furthermore, bimanual intervention focused on child-identified goals may facilitate progress on functional activities within a contextually relevant framework that includes dual roles of the hands to stabilize and manipulate objects depending on the task requirements [18]. Prior investigations suggest that children with UCP demonstrate functional gains following bimanual intervention which are comparable to other upper limb rehabilitation approaches such as constraint-induced movement therapy [19, 20].

To optimize the efficacy of rehabilitation, training may be paired with transcranial direct current stimulation (tDCS), a form of interventional NIBS. TDCS has polarity-specific effects on cortical excitability: anodal is associated with excitatory after-effects, and cathodal is associated with inhibitory after-effects [21]. The pairing of rehabilitation with NIBS has the potential to mitigate maladaptive neuroplasticity and promote the optimal neurophysiologic state for recovery in individuals with stroke [22]. For instance, cathodal tDCS targeting the nonlesioned primary motor cortex paired with constraint-induced movement therapy can rebalance the excitability of both hemispheres and the changes in cortical excitability seen were associated with changes in function [23]. However, a recent study suggested that children with contralateral CST circuitry demonstrated greater benefit from combined intervention than did children with ipsilateral CST circuitry [15].

The purpose of this study was to explore the influence of bimanual intervention paired with cathodal tDCS to the nonlesioned hemisphere on behavioral and neurophysiologic outcomes in children with UCP. We hypothesized that cathodal tDCS will decrease excitability of the nonlesioned hemisphere and pairing with bimanual training will promote the synergistic plasticity between the hemispheres through enhanced sensorimotor integration of information, leading to increased excitability of the lesioned hemisphere. To further examine the effect of underlying CST circuitry patterns on the response to intervention [10, 15], and to minimize heterogeneity in our sample, we focused on children within the contralateral CST circuitry continuum.

## 2. Materials and Methods

### 2.1. Design

**Figure 1:**
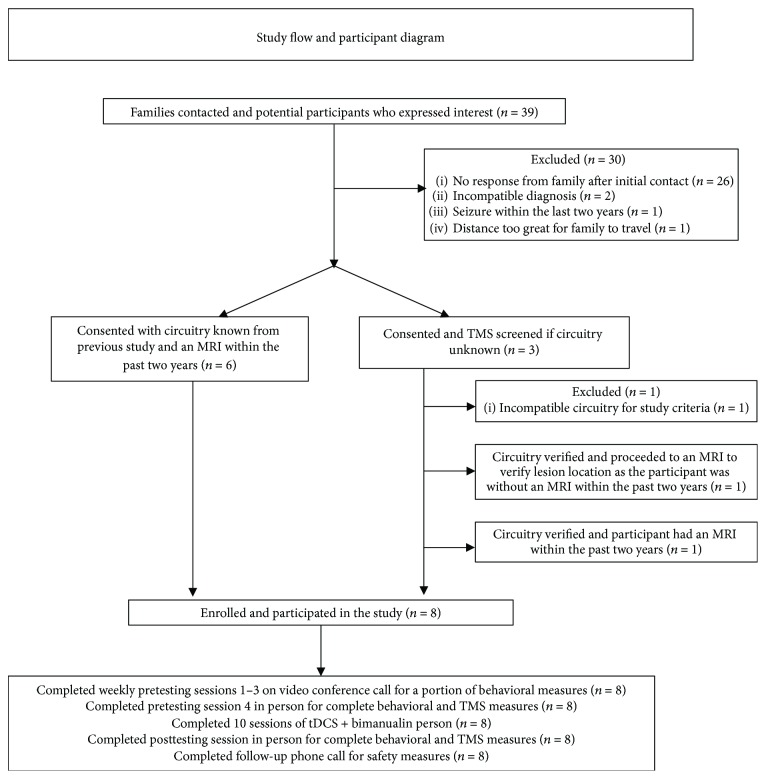
Study flow and participant diagram. MRI: magnetic resonance imaging; tDCS: transcranial direct current stimulation; TMS: transcranial magnetic stimulation.

A single-subject, multiple-baseline, open-label study in children and young adults with UCP was conducted. Changes in behavioral and neurophysiologic outcomes after a 10-day active tDCS + bimanual intervention were compared to their individual baseline performance in the absence of a control group. Each child completed 4 baseline sessions including behavioral and self-reported measures. To meet the needs of participants throughout a wide geographical region, pretesting sessions #1–3 were conducted with real-time video conference calling. Pretesting #4, all intervention sessions, and the posttest were completed in person at a university laboratory (Figure 1). All behavioral testing was completed prior to TMS testing.

### 2.2. Participants

Children and young adults ages 7–21 (mean 13 years, 3 months ± 3.7 years) with imaging-confirmed perinatal stroke were recruited for this study using a laboratory database of past study participants and recruitment of new participants through physician referrals. Inclusion criteria required the presence of a MEP from both hemispheres as assessed by TMS (i.e., children with contralateral CST or bilateral CST circuitry). Exclusion criteria included seizures within the past two years, implanted metal or medical devices contraindicated for NIBS testing or interventions, co-occurring disorders or medical condition (e.g., brain injury, neoplasm, and pregnancy), communication deficits preventing the answering of safety questions, or a history of phenol or botulinum toxin injections within the past 6 months [24, 25].

This study was approved by the University of Minnesota's Institutional Review Board. All participants ages 18 years and older and caregivers of children ages 7–17 provided consent after informed consent discussion. All children ages 7–17 provided assent. This study was registered on clinicaltrials.gov (NCT02250092).

### 2.3. Outcome Measures

#### 2.3.1. Safety Measures

Safety was monitored with surveys, caregiver input, vital sign assessment, and grip strength tests. Assessment of safety occurred before and after all brain stimulation (e.g., TMS testing and tDCS sessions) [26]. An independent medical monitor reviewed all safety data.

#### 2.3.2. Hand Function Measures

Hand function was assessed with performance and self-report measures. The primary outcome was the Assisting Hand Assessment (AHA). The AHA is a measure of spontaneous, bimanual hand function [27]. A change of five AHA units is the smallest detectable difference (SDD) [28]. A rater blinded to the testing session (i.e., pretest or posttest) scored the AHA videos.

Secondary behavioral outcomes included the Box and Blocks, Canadian Occupational Performance Measure (COPM), and the ABILHAND-KIDS. Self-reported measures incorporated participant and caregiver feedback. The Box and Blocks is an assessment of gross unimanual dexterity, and the average of three trials is reported to the nearest integer (Performance Health, Warrenville, IL, USA). The COPM is an occupation-centered, child-rated goal-setting measure with a scale of 1–10 (1—lowest, 10—highest) of activities of daily living skills [29]. A change of two points represents a clinically important difference [30]. Prior investigations have established reliability of the COPM with parent-proxies; however, the reliability of repeated COPM ratings in children is unknown [31]. In this study, previous ratings were not reviewed prior to the child self-assessed rating at each testing time point. The ABILHAND-KIDS is a 21-item caregiver-reported measure of perceived manual abilities in children with CP [32]. The ABILHAND-chronic stroke version was used for participants over age 16 [33]. The total ABILHAND score was converted to a linear measure of manual ability using logits. The least measurable difference for the ABILHAND is 0.19 logits [32, 33].

#### 2.3.3. TMS Measures

Neurophysiological changes were assessed using single-pulse TMS including motor threshold, MEP amplitude, cortical motor mapping, and cortical silent period (CSP) as indices of cortical excitability. TMS testing was completed within 1 week prior to intervention and within one week following the completion of the tDCS + bimanual intervention. Neurophysiologic responses were assessed with TMS using a 70 mm coil using a Magstim 200 stimulator (Magstim Company Ltd., Dyfed, United Kingdom). TMS methods are previously described in other publications [34, 35]. Briefly, bilateral electromyography data was monitored in real time and stored in a laptop computer using a customized LabVIEW program (v2012, National Instruments, Austin, Texas, USA) for offline analysis using a custom Matlab program (MathWorks, Natick, Massachusetts, USA). The primary muscle of interest was the first dorsal interosseous. Stereotactic neuronavigation (Brainsight, Rogue Research, Quebec, Canada) was used to guide TMS coil placement based on individual neuroanatomy acquired from previous MRI. All participants were positioned in a semireclined chair (Rogue Research, Montreal, Canada) with a custom-made tray (Gillette Lifetime Specialty Healthcare, St. Paul, MN) for consistent positioning of the upper extremities during TMS assessment.

To characterize the clinical population of participants with UCP, comprehensive TMS testing included motor threshold assessment (resting motor threshold (RMT) or, if an RMT was not present, an active motor threshold (AMT)), single-pulse TMS testing (10 analyzable trials) using 120% RMT or 110% AMT testing intensity, and CSP (10 analyzable trials) using 120% RMT. Single-pulse testing verified the presence or absence of a MEP from each hemisphere. The 10 trials of single-pulse testing were assessed with peak-to-peak amplitude.

CSP testing provided a measure of motor cortical inhibition [36]. Variations in CSP duration are observed in adults with stroke and other neurological conditions [36]. The CSP testing protocol involved the participant maintaining a tonic contraction of 20% maximum voluntary contraction of the first dorsal interosseous followed by single-pulse TMS using an intensity of 120% RMT to the hemisphere contralateral to the hand maintaining the contraction. Visual feedback of muscle activity and the 20% maximum voluntary contraction target level was provided. The CSP was measured using methods previously described by Garvey et al. [37]. The onset/offset was calculated with a custom Matlab (MathWorks Inc., Natick, MA) program based on Garvey et al. [37], and all trials were visually inspected.

TMS-derived motor mapping measured the cortical representation of an individual muscle. The motor mapping protocol used an intensity of 110% RMT with 1–5 trials performed at each grid point guided by stereotactic neuronavigation [34]. Grid points and corresponding cortical locations were constructed using four concentric circles (radius 10 cm, 7.9 mm between adjacent points) centered on the motor hotspot (Brainsight, version 2.3.4), producing a map with 81 total grid points. Counts of mapping sites with a MEP response of ≥50 *μ*V are reported.

Motor maps were rendered using a predetermined algorithm within the stereotactic neuronavigation software (Brainsight, version 2.3.4). Specifically, MEP amplitudes measured during the motor mapping assessment were projected onto the individual participant's grid points and transformed to a color map. Each MEP response data point is associated with the position and orientation of the TMS coil. Prior investigations of motor mapping in children with UCP suggest stability of maps between testing sessions in the absence of intervention and indicate that a 20% change in the number of responsive sites following rehabilitation is considered significant [34].

The number of pulses was tracked using stereotactic neuronavigation with a protocol upper limit of 300 pulses per hemisphere. A protocol limit of 85% maximum stimulator output was used for all testing procedures to maximize comfort to the participant. Participants were assessed with a caregiver present.

During the data analysis phase, individual circuitry patterns were identified. Participants displaying contralateral MEPs with no measurable bilateral electromyographic activity after stimulation were described as having *contralateral CST circuitry* [12, 38]. Participants with a MEP from the more affected hand following stimulation to the lesioned hemisphere and bilateral MEPs following stimulation to the nonlesioned hemisphere were classified as having *bilateral CST circuitry*.

### 2.4. Intervention

Ten sessions of tDCS + bimanual intervention occurred in a group model over two weeks (Figure 1). Each participant was paired 1 : 1 with the same trained volunteer interventionist for each session. Intervention sessions were 120 minutes of motor training with the first 20 minutes including simultaneous tDCS. Motor training focused on the participant's goals. At the conclusion of the study, all participants and families were surveyed for study satisfaction.

The 20 minutes of 1.5 mA cathodal tDCS targeted the primary motor cortex of the nonlesioned hemisphere (Soterix 1×1 Limited Total Energy (LTE), New York, NY). Medical grade electrode sponges of 5 × 7 cm with a 25 cm^2^ rubber electrode were used. The center of the cathode electrode sponge was placed on the TMS-derived motor hotspot of the nonlesioned hemisphere. The reference electrode was placed on the contralateral supraorbital region. The location of the TMS-derived motor hotspot was marked daily after tDCS session using a nontoxic marking pen (i.e., Sharpie marker).

### 2.5. Statistical Analysis

To determine the score used for baseline, we first evaluated the reliability of behavioral measures. The reliability of repeated pretest measures was assessed with a one-way analysis of variance and the appropriate intraclass correlation coefficient (Supplemental Table 1). Intraclass coefficients of ≥0.90 reflect excellent reliability, ≥0.75 demonstrates good reliability, ≥0.50 indicates moderate reliability, and <0.50 indicates poor reliability [39]. Using these established intraclass coefficient ranges, the reliability of repeated baseline measures indicated moderate to excellent reliability in the ABILHAND and COPM-Performance subscale and poor reliability in the COPM-satisfaction subscale and the Box and Blocks (Supplemental Table 1).

To evaluate for change following intervention, single-subject analysis involved review of the participant's magnitude of change relative to clinically meaningful changes for each behavioral outcome. The statistic used to determine the clinically meaningful change varied for each behavioral measure. Previously published SDD, minimal clinically important difference (MCID), least measurable difference, and standard error of the mean (SEM) were used for the AHA [28], COPM [40], ABILHAND [32], and the Box and Blocks, respectively. In the absence of a published meaningful difference for the Box and Blocks, the SEM calculated from the multiple pretests in this study was used. If there were multiple baselines of a behavioral measure, we used the average for pre-/post-comparisons. For a single baseline measure, this testing time point was used for comparisons. Magnitude of change was calculated with net change scores, comparing the average of all pretest measures to the posttest score [41]. We also performed a responder analysis of the behavioral outcome measures, where a responder achieved at least the SDD of 5 points on the AHA. No formal analyses were conducted within groups due to sample size. Statistical analyses were conducted using SPSS (IBM, Armonk, NY) and GraphPad Prism (GraphPad Software, La Jolla, CA).

## 3. Results

Nine children enrolled in this study (Figure 1). One participant was excluded following preintervention TMS testing due to the absence of bilateral or contralateral circuitry resulting in a final sample of eight undergoing the pretesting, intervention, and posttesting. Baseline characteristics are reported in Table 1. Structural T1 magnetic resonance images displaying the participant's lesion are provided in the supplemental materials (Supplemental Figure 1).

Structural T1 imaging, data on birth history, age at first imaging, and age at research imaging are provided in supplemental materials (Supplemental Table 3).

### 3.1. Safety Measures

All enrolled participants completed the study without any serious adverse events. In this study, 37.5% (3 participants) reported minor adverse events related to active tDCS in more than one tDCS session, with the most common symptom being unusual feelings on the skin of the head (Supplemental Table 2). Three participants experienced spasms in their more affected hand during tDCS + bimanual intervention. All reported symptoms resolved within the same session. Overall, a small proportion (12–37%) of individuals reported tDCS-related minor adverse events suggesting that the tDCS intensity of 1.5 mA was well tolerated by participants.

#### 3.1.1. Individual Analysis of Meaningful Change

**Figure 2:**
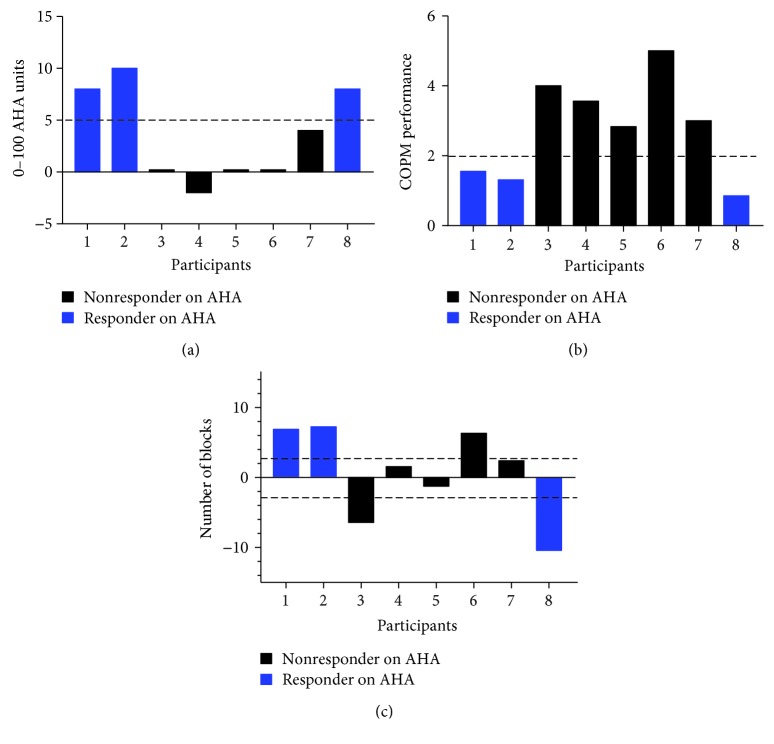
Individual change in behavioral measures (a) AHA with the SDD denoted with a dashed line. (b) COPM-Performance. The MCID of the COPM is denoted with a dashed line. (c) Box and Blocks with more affected hand. The SEM of repeated baseline testing with the Box and Blocks is denoted with a dashed line. AHA: Assisting Hand Assessment; COPM: Canadian Occupational Performance Measure; MCID: minimal clinically important difference; SDD: smallest detectable difference. Note: the *y*-axis representing change differs between the measures. Blue bars represent participants identified as a responder on the primary outcome (AHA), and black bars represent participants identified as a nonresponder on the primary outcome (AHA).

All participants achieved clinically meaningful change on at least one measure (AHA, COPM-Performance and/or Satisfaction, and ABILHAND). Individual changes on behavioral measures are reported in Table 2, and individual performance measures over all testing sessions are provided in the supplemental data (Supplemental Figure 2). Changes on each of the behavioral measures based on the responder analysis are reported in Figure 2.

Collectively, changes in behavioral measures varied for participants in this study including bimanual, unimanual, self-report, and general study satisfaction. For the primary outcome measure, the AHA, 3 of 8 participants achieved the SDD. Increases on the Box and Blocks that exceeded the SEM were observed in 2 participants for the more affected hand and 1 participant for the less affected hand. For the self-reported measures, 5 of 8 participants achieved the MCID for the COPM and 3 of 8 participants achieved the least measurable difference on the ABILHAND. Of the 3 participants who achieved the SDD on the AHA, none of them achieved the MCID on the COPM. For this open-label study, families reported an average satisfaction rating of 9.5 out of 10 related to their study experience.

#### 3.1.2. TMS Measures

Four participants completed full TMS testing on all measures. In the remaining 4 participants, data collection was limited by the presence of an AMT only, machine intensity exceeding protocol limit of 85% maximum stimulator output, and limited tolerance for lesioned hemisphere testing at posttest. No participants reported use of centrally acting medications for seizure control that would influence neurophysiologic responses.

**Figure 3:**
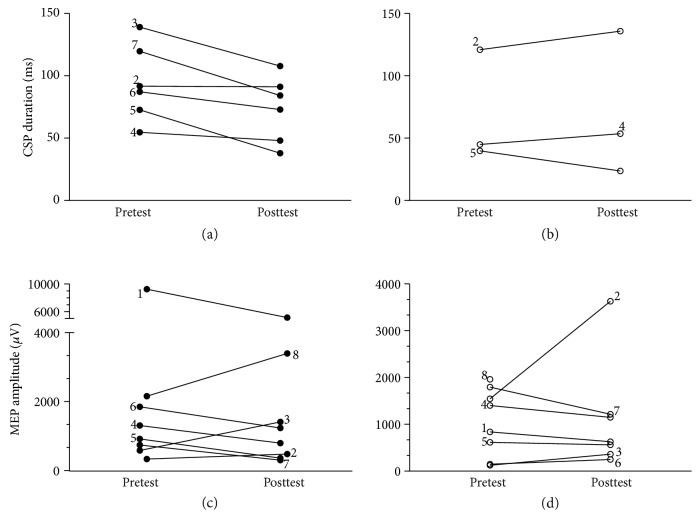
Pre- and posttest neurophysiologic measures. (a) Nonlesioned hemisphere amplitude with single-pulse TMS testing. (b) Lesioned hemisphere amplitude with single-pulse TMS testing. (c) Nonlesioned hemisphere CSP duration. (d) Lesioned hemisphere CSP duration. Nonlesioned data is denoted by a closed circle, and lesioned data is denoted by an open circle. Note: data points are labeled with a superscript participant identifier consistent with Table 1 (participant IDs 1–8). The *y*-axis representing change differs between the measures. CSP: cortical silent period; ms: milliseconds; SP: single-pulse; TMS: transcranial magnetic stimulation. Amplitudes are measured in *μ*V (microvolts).

Single-pulse amplitude was measured in 8 of 8 participants on the nonlesioned hemisphere with hypothesized decreases observed in 5 of 8 children. The nonlesioned hemisphere CSP duration was measured in 6 of 8 children and all exhibited decreases from pretest to posttest. Lesioned hemisphere single-pulse amplitude was measured in 7 of 8 children with hypothesized increases observed in 3 children. Lesioned hemisphere CSP duration was measured in 3 of 8 children with increases observed in 2 children. No statistical analyses were conducted on cortical excitability data due to small sample. Individual changes in MEP amplitude and CSP duration are shown in Figure 3.

**Figure 4:**
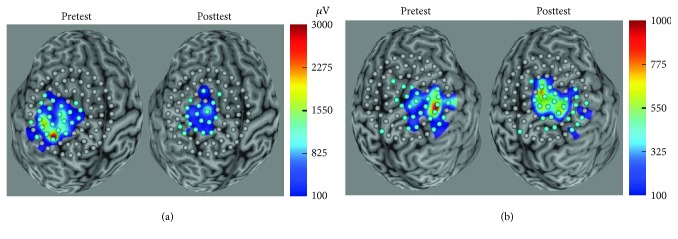
TMS-derived motor map of (a) nonlesioned and (b) lesioned hemispheres of participant 2 with the grid centered on the TMS-derived motor hotspot. The brain reconstruction has been rotated to allow for a direct view of the TMS-derived motor hotspot. Grey grid points signify no MEP responses, and teal grid points signify MEP responses > 50 *μ*V (microvolts). The color bar represents the range of amplitude of MEP responses in *μ*V. The range in the amplitude color bar is dependent on the magnitude of the responses observed. Note: ranges are consistent across pre- and posttesting sessions but may differ across hemispheres. CST: corticospinal tract; MEP: motor-evoked potential; TMS: transcranial magnetic stimulation.

TMS motor map data were collected on 6 participants; 4 of 6 had mapping data for both hemispheres. Hemispheric differences (lesioned vs. nonlesioned) appeared to influence changes in cortical mapping with variations in map features observed. The lesioned hemisphere cortical map sites decreased in 3 participants and increased in 1 participant (Table 3). The nonlesioned hemisphere cortical map sites increased in 4 participants and decreased in 2 participants (Table 4). In the 4 participants who have cortical maps for both hemispheres, the changes in response sites for one hemisphere resulted in an inverse change in the opposite hemisphere (e.g., if the lesioned hemisphere increased in number of responsive sites, the nonlesioned hemisphere decreased in number of responsive sites).  Figure 4 displays map changes in one representative participant with mapping data collected from both hemispheres.

Exploratory correlations were conducted between behavioral and neurophysiologic outcomes. There was a strong correlation observed between baseline AHA score and the motor threshold of the lesioned hemisphere at baseline (Spearman's rho correlation coefficient: −0.71, *p* = 0.05) (Supplemental Table 4). All other correlations between baseline neurophysiologic measures and change in behavior were nonsignificant (*p* > 0.05).

## 4. Discussion

### 4.1. Confirming Safety Results

Serial sessions of combined active tDCS + bimanual intervention were safe and feasible in children with UCP. In our study, the most common minor adverse events were unusual feelings on the skin of the head, and symptoms were mitigated using study protocols. For participants who experienced spasms, alternating fine and gross motor activities were found to be beneficial in reducing the occurrence of a spasm during the same session.

### 4.2. Variable Gains in Hand Function and Self-Reported Measures

For this study, we identified “responders” to tDCS based on changes on the AHA. In this sample, three participants (37.5% of sample) achieved the SDD (5 points) on the AHA following the 20 hours of combined intervention. Others have reported that 30% of participants achieved the SDD on the AHA following 90 hours of bimanual intervention alone [16]. Of the participants identified as a responder, two displayed contralateral circuitry and one displayed bilateral circuitry. In this subgroup of responders, we observed a wide range of baseline AHA scores (34 AHA units to 83 AHA units), age (8 to 14 years old), and both types of circuitry patterns.

The single-case design of our study also allowed for individual analysis which may allow us to generate hypotheses for larger studies and may be preferred over group analyses that mask sensitivity of changes by evaluating group mean response. For example, participants 1 and 2 could be considered strong candidates for tDCS as they both displayed contralateral circuitry and high AHA scores at baseline. Both of these participants demonstrated further improvements following intervention on bimanual (AHA) and unimanual (Box and Blocks) measures. The improvements suggest that these participants had the ability to differentiate roles of the hands for bimanual tasks, and further improvements on the Box and Blocks may reflect a training benefit to each hand. In contrast, participant 8 displayed lower bimanual hand function at baseline and achieved the SDD on the AHA following intervention but did not demonstrate unimanual gains on the Box and Blocks. This suggests that this participant may have significantly benefitted from bimanual training alone. These preliminary data contribute to our understanding of who might benefit most from combined interventions or motor training alone which could be assessed in future studies that could guide personalized medicine approaches for stroke rehabilitation.

Our behavioral measures were selected to reflect observed performance of bimanual (AHA) and unimanual (Box and Blocks) hand function, the child's perception of goal attainment (COPM), and the caregiver's perception of hand use (ABILHAND). Each of these assessments contributes to a broader understanding of how combined interventions impacted hand function in this sample of eight children. No one pattern of change was desired as the baseline behavioral function of each child varied.

From a behavioral standpoint, the motor learning resulting from the intervention may have been highly task-specific with changes in motor function not observed in general movements captured on the primary outcome measure, the AHA. The AHA is a measure of how the participant chooses to use his/her hand during a novel task whereas the COPM is a self-reported measure of the participant's perspective of their achievement of goals regardless of how the participant is able to accomplish the goal (e.g., compensatory vs. newly acquired motor movements). Therefore, the construct underlying each of the behavioral measures may have differed providing a comprehensive assessment of the intervention effects on physical abilities for desired activities.

Variability in the Box and Blocks was observed during multiple baseline testing sessions and with average pretest/posttest comparisons. Decreases observed in the more affected hand, measured with the Box and Blocks, could be attributed to sensitivity of the measure or engagement in testing procedures. Others have used the Box and Blocks as a daily measure of safety with results suggesting that individual variability is observed in children with UCP [42]. This evidence warrants continued monitoring for a decrement in unimanual dexterity in combined NIBS and motor training studies with larger samples.

The assessment tools selected for this study when taken together represent components of bimanual and unimanual hand function reporting both on the impairment and activity levels of the World Health Organization's International Classification of Functioning, Disability, and Health framework. The individual variability in gains observed between the assessment tools reflects differing constructs for each measure as reported by others [43].

### 4.3. Neurophysiologic Influences

The effects of tDCS can be evaluated through changes in cortical excitability using single-pulse TMS [21]. Varying neurophysiologic effects of the combined tDCS and bimanual intervention were observed in the nonlesioned hemispheres as measured by TMS. We observed changes in the measures of amplitude of the nonlesioned hemisphere in five of eight participants consistent with a hypothesized decrease in excitability after the tDCS + bimanual intervention. In contrast, a shortening of CSP duration in six of six participants and an increase in the number of mapping sites in four of six participants with complete data suggests an increased excitability in the nonlesioned hemisphere. From a neurophysiologic standpoint, the interindividual variability observed in this study could be attributed to known factors (e.g., anatomical differences and lesion locations) and factors unexamined in this study (e.g., the influence of maturation on brain development, genetics, functional organization of circuits, baseline neurophysiologic state, and capacity for change in motor learning) that collectively may influence plasticity [44, 45].

Prior reports have shown that MEP amplitude changes are dependent on tDCS polarity (e.g., anodal stimulation results in increased MEP amplitude) [21]. However, the duration of stimulation and the pairing of stimulation with a cognitive task may result in paradoxical effects, such as cathodal tDCS producing increased MEP amplitude [46, 47]. The combination of bimanual activity and stimulation could have produced similar unexpected changes in cortical excitability and explain the variability observed in MEP amplitude and motor mapping data.

We did not measure IHI directly; however, our CSP measurements suggest decreased intracortical inhibition. Because exaggerated IHI may not be present in all children with injury early in life, a comprehensive understanding of both activity-dependent withdrawal and the balance of IHI on CST development and resultant hand function is needed to understand potential response to rehabilitation interventions [6]. Future longitudinal studies with multimodal outcomes will elucidate the neurophysiologic substrates of change following combined interventions in children and young adults with UCP.

Similar conflicting results between neurophysiologic measures were observed in the lesioned hemisphere. Three participants displayed increased excitability (e.g., a decrease in motor threshold and increase in MEP amplitude) whereas three participants displayed a mixed response to stimulation (e.g., decreased motor thresholds and decreased amplitudes).

Our motor mapping results reflect both expanded and reduced map size on each hemisphere, which differs from prior reports of map expansion in response to motor training in both animal models and human studies. The changes in motor maps suggest that cortical map changes are activity-dependent [34, 48, 49]. These results strengthen the argument for an activity-dependent competition model of neuroplastic change in children with UCP [3]. Recent rehabilitation studies have examined motor map changes after bimanual, occupation-focused intervention reporting bilateral increases in map size and amplitude of responses and no significant change in motor threshold [50] and increases in the number of responsive sites following bimanual intervention in children with UCP [34]. In our study, the changes in the number of responsive sites may reflect a broader intrahemispheric network as our grid encompassed a region beyond primary motor cortical area, but responses outside this region warrant further confirmation in future studies.

### 4.4. Lesion Location and Clinical Presentation May Influence Variability in Outcomes

In our sample, two participants with a right-sided lesion demonstrated similar patterns of change in neurophysiologic responses in the nonlesioned hemisphere as well as similar change in behavior. The heterogeneity of changes observed in participants with left-sided lesions may be attributed to the potential for hemispheric crowding of function suggesting that reorganization of function to the right hemisphere following a left hemisphere lesion may have a negative impact on typical functions of the right hemisphere [51–53]. Studies with larger samples may allow investigators to discern the association of lesion side to response to intervention in children with UCP.

Lesion location could have influenced the results; however, we are unable to determine this potential with our sample size. Our study represents a clinical sample of individuals with UCP where 6 of the 8 participants displayed combined cortical and subcortical lesions and the remaining 2 participants displayed subcortical lesions only. None of our participants presented with documented bilateral lesions. However, we cannot rule out that the lesion could have influenced the contralateral hemisphere, which may not be noted in the MRI report. We did observe a relationship between single-pulse motor-evoked potential amplitude of the nonlesioned hemisphere and baseline AHA score, but no correlations existed when evaluating changes among neurophysiologic and behavioral measures. One potential explanation is that compensatory descending tracts may be involved in recovery, warranting expanded investigations.

### 4.5. Limitations

Our conclusions are limited by the sample size, the open-label study design, and the sensitivity of our measures, and as such, the findings should be considered preliminary. Our study was open-label which may have influenced the self-reported measures of change and satisfaction by both participants and caregivers. This study design was selected to explore preliminary findings and provide direction for future studies. The lack of control group is limitation and must be considered when interpreting the findings.

We did not have an immediate measure of cortical excitability following tDCS (e.g., within minutes of completing tDCS). An immediate measure could evaluate not only reliability of single-pulse measures in children with UCP but also the time-effect of observed changes in neurophysiologic responses. For this study, the duration of participation encompassing daily safety measures and motor intervention precluded any additional testing.

In our study, motor training was individualized to the child's goals allowing for impairment-specific intervention (e.g., increasing the distance a child can reach with the more affected limb) in order to improve activity-specific performance (e.g., pushing the more affected limb through a t-shirt sleeve). This form of personalized intervention positively impacted self-reported goal achievement but may have influenced the effects of tDCS on other measures. Additionally, providing the intervention in a group format could have influenced the engagement of the participants, reports of side effects, and the caregiver and/or participant's perspective of change as self-reported on behavioral measures.

### 4.6. Future Directions

Future personalized medicine will allow for interventions to be optimally paired for children based on biomarkers or clinical practice guidelines given the child's clinical presentation. Critical areas of need in the pediatric investigations include computational modeling to optimize electrode placement, expanded testing (behavioral, neurophysiologic, and neuroimaging), and defined motor training protocols.

Prior investigations suggest that the peak electric fields are higher in children as compared to adults when modeled at the same tDCS intensity [54, 55]. Studies that incorporate computational modeling could allow for individualized stimulation parameters and may assist in controlling for anatomical differences between participants. For example, further comparison of electrode placement based on individual circuitry such as using anodal stimulation to the hemisphere of greatest control of the more affected hand (e.g., participants with contralateral circuitry may benefit from anodal stimulation to the lesioned hemisphere as compared to targeting the nonlesioned hemisphere in participants with ipsilateral circuitry). Electrode placement taken into consideration with comparative effectiveness studies on session frequency and duration of combined intervention in differing CST circuitry subtypes (e.g., greater number of sessions or a longer duration) could provide guidance for treatment protocols in the future. Further, it may be that alternate electrode placements (e.g., premotor area) not previously studied in children with UCP are more efficacious than the primary motor cortex as suggested in the adult literature [56].

Future studies with larger samples and expanded testing protocols will inform our knowledge of the mechanism of tDCS on motor learning and the aspects of motor learning that can be targeted with combined neuromodulatory interventions. Our performance and self-reported behavioral measures may not be sensitive enough to detect changes in neurophysiologic responses. Expanded testing protocols could focus on sensorimotor input including isolated movements and sensory function which may have stronger associations to neurophysiologic changes [57, 58]. Further, daily motor learning curves captured by a kinematic measure of hand function may allow for individualization of intervention [59]. Altogether, these measures may identify the sensorimotor contributions to change in function following intervention [60]. Expanded testing will assist in identifying which pediatric measures have the greatest sensitivity for measuring change following combined neuromodulatory and rehabilitation interventions.

Defining the critical components of the motor training and standardizing the motor training components (e.g., percentage of time spent on unimanual vs. bimanual tasks, types of tasks and motor movements targeted, and home program tasks) will allow for investigators to control for these potential influences when measuring for the effects of tDCS. Prior studies report that tasks with cognitive challenges, isometric contractions, and the duration of noninvasive brain stimulation can influence the observed neurophysiologic changes in response to tDCS [46, 47, 61]. Studies designed with an immediate neurophysiological measure following tDCS can provide insights into pairing activity with stimulation in children with UCP and potential paradoxical effects when pairing tDCS with a motor task.

## 5. Conclusion

The combined intervention of tDCS with occupation-centered rehabilitation intervention was safe and well-tolerated. Participants identified and rated their performance on goals that were meaningful to them, and customized activities during combined tDCS and bimanual training sessions designed to promote goal achievement proved feasible in a group setting. Our neurophysiologic findings suggest that the combined intervention affects each hemisphere differently, which may underlie variability observed with changes in behavioral hand function measures. A consistent neurophysiologic substrate that influences response to change has not yet been identified in children with UCP. Future clinical trials should consider cortical excitability evaluations based on underlying circuitry to measure changes following interventions, which may elucidate neurophysiologic mechanisms related to recovery.

## Figures and Tables

**Table 1:** Participant characteristics and function.

ID	Age (y)	Sex	Lesion location	Lesion details	Affected side	MACS level	CST circuitry	Lesioned MT	Nonlesioned MT
1	8	M	Cortical and subcortical	Left MCA distribution, frontal and parietal operculum, left PLIC, cerebral peduncle, and ventral pons	R	II	Contralateral	52	46
2	14	F	Subcortical	Left lateral ventricle, centrum semiovale, and internal capsule	R	II	Contralateral	44	41
3	14	F	Cortical and subcortical	Left lateral ventricle with adjacent thinning of cortex and corpus callosum	R	III	Contralateral	64	57
4	10	F	Subcortical	Left lacunar infarct in thalamus	R	II	Bilateral	66	63
5	15	F	Cortical and subcortical	Left MCA distribution, frontoparietal cortex, left thalamus, and basal ganglia	R	I	Bilateral	46	42
6	19	F	Cortical and subcortical	Right lateral ventricle and posterior right frontal lobe	L	II	Bilateral	44	38
7	12	M	Cortical and subcortical	Right thalamus and periventricular white matter	L	II	Bilateral	75	54
8	8	M	Cortical and subcortical	Left frontal lobe and posterior parietal lobe; left subinsular, caudate, and lentiform nuclei; left basal ganglia and hypothalamic region; and left cerebral peduncle	R	III	Bilateral	77	48

CST: corticospinal tract; F: female, L: left; M: male; MACS: Manual Ability Classification System; MCA: middle cerebral artery; MT: motor threshold; PLIC: posterior limb of internal capsule; R: right; y: years. Lesion location was identified by a pediatric neurologist as cortical, subcortical, or both cortical and subcortical. CST circuitry pattern was identified with single-pulse transcranial magnetic stimulation testing.

**Table 2:** Participant function and behavioral change scores.

ID	Circuitry	AHA	AHA ∆	Box and Blocks MA	Box and Blocks MA ∆	Box and Blocks LA	Box and Blocks LA ∆	COPM-Perf	COPM-Perf ∆	ABILHAND	ABILHAND ∆
1	Contra	60	**8**	19.08	**7**	57.58	**9**	3.45	1.55	1.48	**0.28**
2	Contra	83	**10**	27.08	**8**	70.33	**15**	3.70	1.31	3.90	0.00
3	Contra	52	0	15.08	**−7**	31.33	1	2.67	**4.00**	0.52	**0.62**
4	Bilateral	54	−2	3.78	2	61.17	−4	4.69	**3.56**	2.02	0.15
5	Bilateral	53	0	12.58	−2	48.33	**10**	3.83	**2.83**	3.80	0.10
6	Bilateral	75	0	25.67	**7**	79.33	3	1.00	**5.00**	0.72	**0.48**
7	Bilateral	55	4	41.25	3	65.92	**7**	5.00	**3.00**	1.79	−0.03
8	Bilateral	34	**8**	0	**−10**	34.92	−2	3.35	0.85	0.25	−0.08

AHA: Assisting Hand Assessment; Contra: contralateral; COPM: Canadian Occupational Performance Measure; LA: less affected hand, MA: more affected hand; ∆: change. AHA is reported in 0–100 AHA units, Box and Blocks is reported as a mean, COPM is reported as a mean, and ABILHAND is reported in logits. Baseline testing for Box and Blocks, COPM, and ABILHAND reflects the average of 4 pretests. The change score is calculated with posttest-average baseline score. Achievement of smallest detectable difference (AHA), least measurable difference (ABILHAND), and clinically meaningful differences (COPM) are denoted in bold. Given the precision of the measurement, change in Box and Blocks that exceeds the standard error of the measure is denoted in bold (>3 blocks for MA hand and >6 blocks for LA hand).

**Table 3:** Lesioned hemisphere cortical excitability mapping measures.

ID	Laterality	Mapping testing intensity (% MSO)	Lesioned hemisphere				
			Pretest FDI sites	Pretest FDI mapping latency (ms)	Posttest FDI sites	Posttest FDI mapping latency (ms)	% ∆ in FDI sites
1	Contralateral	†	†	†	†	†	†
2	Contralateral	48	24	22.50	18	20.19	**−25.00**
3	Contralateral	70	24	27.06	23	25.26	−4.17
4	Bilateral	†	†	†	†	†	†
5	Bilateral	51	39	49.95	57	47.51	**46.15**
6	Bilateral	38	38	58.00	27	58.36	**−28.95**
7	Bilateral	†	†	†	†	†	†
8	Bilateral	†	†	†	†	†	†

FDI: first dorsal interosseous; ID: participant identifier; ms: milliseconds; NA: not assessed at baseline; NC: not calculated due to missing baseline data; RMT: resting motor threshold; ∆: change; †: missing data; % MSO: percentage of maximum stimulator output. Motor mapping testing intensity was 110% RMT (resting motor threshold). MEP latency durations are reported using the mean time (ms). Bolded values are considered significant defined as ≥20% mapping response sites.

**Table 4:** Nonlesioned hemisphere cortical excitability mapping measures.

ID	Laterality	Mapping testing intensity (% MSO)	Nonlesioned hemisphere				
			Pretest FDI sites	Pretest FDI mapping latency (ms)	Posttest FDI sites	Posttest FDI mapping latency (ms)	% ∆ in FDI sites
1	Contralateral	†	†	†	†	†	†
2	Contralateral	45	25	20.47	31	20.47	**24.00**
3	Contralateral	63	27	21.41	32	20.20	18.52
4	Bilateral	69	17	18.65	14	16.73	−17.65
5	Bilateral	46	15	19.51	13	19.19	−13.33
6	Bilateral	42	10	18.06	14	19.20	**40.00**
7	Bilateral	59	15	26.50	23	19.30	**53.33**
8	Bilateral	†	†	†	†	†	†

FDI: first dorsal interosseous; ID: participant identifier; ms: milliseconds; NA: not assessed at baseline; NC: not calculated due to missing baseline data; RMT: resting motor threshold; ∆: change; †: missing data; % MSO: percentage of maximum stimulator output. Motor mapping testing intensity was 110% RMT (resting motor threshold). MEP latency durations are reported using the mean time (ms). Bolded values are considered significant defined as ≥20% mapping response sites.

## Data Availability

The data used to support the findings of this study are restricted due to patient privacy. Access to these data will be considered by the author upon request, with permission from the Institutional Review Board.

## Supplementary Materials

Supplementary Materials Supplemental Table 1: reliability of pretesting measures. Each participant completed repeated baseline behavioral testing measures (1x/week for four weeks) as a part of this study protocol. This supplemental material includes the reliability data of the repeated pretesting measures and an interpretation of the reliability data. Based on this reliability data, we decided to use an average of the four baseline pretesting scores as the comparator for pre- and postintervention comparisons. Supplemental Table 2: tDCS-related MAE. Supplemental Figure 1: axial images of T1 anatomical magnetic resonance image (MRI) to display lesion location in each participant. Row one of the images reflects participants 1–4, and row two reflects participants 5–8. Verification of diagnosis was a criterion for inclusion for this study. T1 anatomical images were collected either (1) at the time of study participation or (2) within 2 years of study participation. The age range of participants at the time of imaging for this study was 8 years, 1 month to 18 years, and 1 month. A pediatric neurologist verified the lesion location. All MRI data was acquired using a 3 Tesla MRI scanner using a 32-channel head coil (Siemens Prisma scanner). Supplemental Table 3: birth and imaging history of participants. Supplemental Figure 2: change in behavioral measures repeated over time. All participants completed repeated baseline measure of behavioral measures including Canadian Occupational Performance Measure-Performance, ABILHAND, Box and Blocks with each hand, and Assisting Hand Assessment. These data represent individual data for repeated baseline measures and posttesting. Supplemental Table 4: Spearman's correlation coefficient between neurophysiologic and hand function measures at baseline.
