# Supplementary material for: Transcranial Direct Current Stimulation (tDCS) Paired with Occupation-Centered Bimanual Training in Children with Unilateral Cerebral Palsy: A Preliminary Study
Source: Neural Plast. 2018 Nov 5;2018:9610812. doi: 10.1155/2018/9610812 (PMC6304908; doi:10.1155/2018/9610812)
Supplement: Supplementary Materials — Supplemental Table 1: reliability of pretesting measures. Each participant completed repeated baseline behavioral testing measures (1x/week for four weeks) as a part of this study protocol. This supplemental material includes the reliability data of the repeated pretesting measures and an interpretation of the reliability data. Based on this reliability data, we decided to use an average of the four baseline pretesting scores as the comparator for pre- and postintervention comparisons. Supplemental Table 2: tDCS-related MAE. Supplemental Figure 1: axial images of T1 anatomical magnetic resonance image (MRI) to display lesion location in each participant. Row one of the images reflects participants 1–4, and row two reflects participants 5–8. Verification of diagnosis was a criterion for inclusion for this study. T1 anatomical images were collected either (1) at the time of study participation or (2) within 2 years of study participation. The age range of participants at the time of imaging for this study was 8 years, 1 month to 18 years, and 1 month. A pediatric neurologist verified the lesion location. All MRI data was acquired using a 3 Tesla MRI scanner using a 32-channel head coil (Siemens Prisma scanner). Supplemental Table 3: birth and imaging history of participants. Supplemental Figure 2: change in behavioral measures repeated over time. All participants completed repeated baseline measure of behavioral measures including Canadian Occupational Performance Measure-Performance, ABILHAND, Box and Blocks with each hand, and Assisting Hand Assessment. These data represent individual data for repeated baseline measures and posttesting. Supplemental Table 4: Spearman's correlation coefficient between neurophysiologic and hand function measures at baseline. [file 9610812.f1.pdf]

SUPPLEMENTAL MATERIALS

Supplemental Table 1. Reliability of Pre-Testing Measures

Test Measure	ICC [LCI, UCI]	SEM[1]
ABILHAND	0.95 [.86, .99]	0.30
COPM-Performance	0.78 [.51, .94]	0.65
COPM-Satisfaction	0.66 [.33, .90]	0.90
Box and Blocks-More-affected hand	0.97 [.93, .99]	2.27
Box and Blocks-Less-affected hand	0.91 [.77, .98]	5.26

[1]SEM units are as follows: ABILHAND: Logit points; COPM: points, Box and Blocks: blocks. CI: Confidence Interval; COPM: Canadian Occupational Performance Measure; ICC: Intraclass correlation coefficient; LCI: Lower bound of 95% Confidence Interval; UCL: Upper bound of 95% Confidence Interval; SEM: Standard error of measurement.

ICCs of ≥0.90 reflect excellent reliability, ≥0.75 demonstrate good reliability, ≥0.50 indicates moderate reliability, <0.50 indicates poor reliability.[39] This means that excellent reliability was found in the ABILHAND and both hands for the Box and Blocks, good reliability was found in the COPM Performance subscale, moderate reliability in the COPM-Satisfaction subscale. For the ABILHAND and COPM-Performance subscale, both measures demonstrated acceptable ICC's, narrow 95% CI and low SEM. The wide 95% CI for the COPM-Satisfaction paired with the lower ICC as compared to the COPM-Performance subscale and larger SEM suggests the measure may not be reliably rated by children. The potential exists that satisfaction as a concept is difficult for children to understand which may have influenced the reliability of weekly baseline ratings. The SEM is largest in the Box and Blocks as compared to other measures with a higher SEM in the less-affected hand as compared to the more-affected hand. This may reflect acquired learning with weekly trials. Given the precision of the Box and Blocks measure, the SEM will be considered 3 blocks for the more-affected hand and 6 blocks for the less-affected hand.

The purpose of multiple baseline assessments was to establish a consistent baseline. Therefore, to be consistent, an average of the 4 baseline assessments was used to represent a stable baseline comparator for paired pre/post comparisons.

Supplemental Table 2. tDCS-Related MAE

	Active tDCS + Bimanual, n=8	
	Proportion of participants reporting MAE	Number of Reports
Unusual feelings on the skin of the head	0.37 (3 participants)	8 reports
Spasm in more-affected hand	0.37 (3 participants)	5 reports
Itchiness	0.25 (2 participants)	2 reports
Tingling	0.25 (2 participants)	3 reports
Sleepiness	0.12 (1 participant)	2 reports
Difficulty paying attention	0.12 (1 participant)	1 report
Headache	0.12 (1 participant)	1 report

tDCS: transcranial Direct Current Stimulation; MAE: Minor Adverse Events.

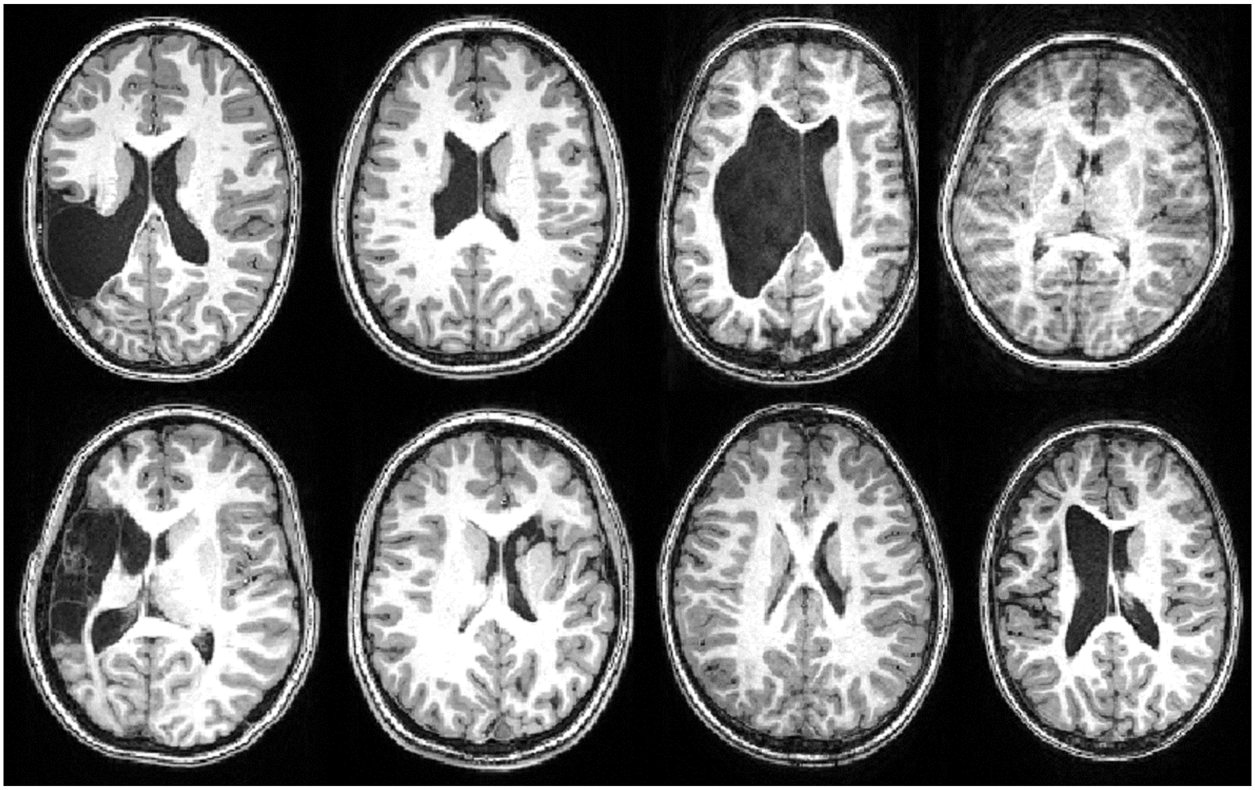

Supplemental Figure 1. Axial images of T1 anatomical magnetic resonance image (MRI) to display lesion location in each participant. Row one of the images reflects Participant 1-4 and row two reflects Participants 5-8. Verification of diagnosis was a criterion for inclusion for this study. T1 anatomical images were collected either 1) at the time of study participation or 2) within 2 years of study participation. The age range of participants at the time of imaging for this study was 8 years, 1 month to 18 years, 1 month. A pediatric neurologist verified the lesion location. All MRI data was acquired at using a 3 Tesla MR scanner using a 32-channel head coil (Siemens Prisma scanner).

Supplemental Table 3. Birth and imaging history of participants.

Participant ID	Birth History	Age at first imaging (yrs, mo)	Age at research MRI (yrs, mo)
1	Full term	0 yrs, 7 days	8 yrs, 1 mo
2	Full term	2 yrs, 5 mo	13 yrs, 5 mo
3	Pre-term	0 yrs, 10 mo	14 yrs, 0 mo
4	Pre-term	0 yrs, 10 mo	9 yrs, 4 mo
5	Full term	0 yrs, 2 mo	14 yrs, 8 mo
6	Full term	0 yrs, 10 mo	18 yrs, 1 mo
7	Full term	2 yrs, 10 mo	10 yrs, 11 mo
8	Pre-term	0 yrs, 9 mo	8 yrs, 7 mo

Mo: months; MRI: Magnetic resonance imaging; Yrs: years.

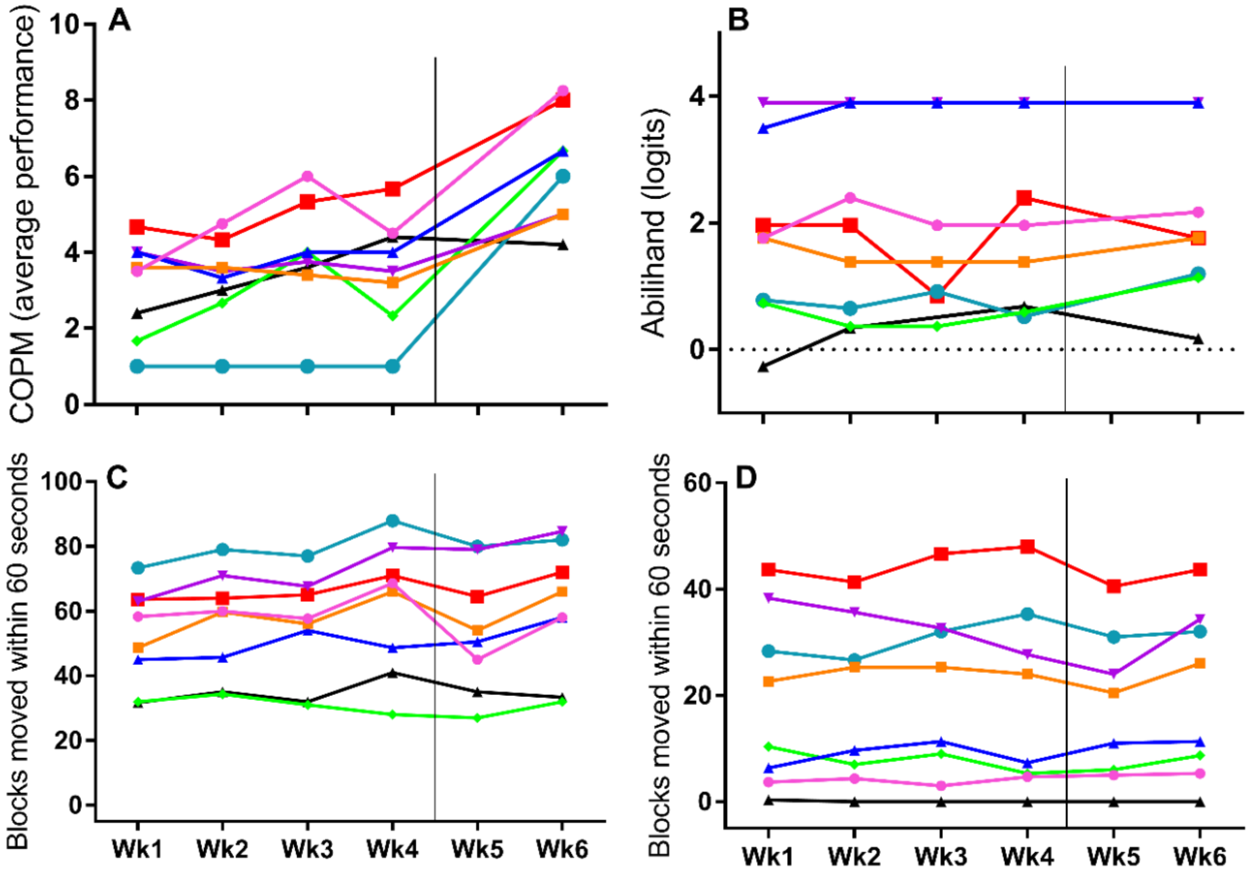

Supplemental Figure 2. Change in behavioral measures repeated over time. For repeated measures testing, each participant is identified in a different color and symbol. Baseline testing sessions are identified as Week 1 (Wk1), Week 2 (Wk2), Week 3 (Wk3), Week 4 (Wk4), Week 5 (Wk5), and post-testing at Week 6 (Wk6). The start of intervention is denoted with a solid black line. (A) COPM-Performance. (B) ABILHAND (C) Box and Blocks with less-affected hand (D) Box and Blocks with more-affected hand. AHA: Assisting hand assessment; COPM: Canadian Occupational Performance Measure. Note: The y-axis representing the range of scores differs between the measures. The Box and Blocks was collected at Wk5 for interim safety testing.

Supplemental Table 4. Spearman's correlation coefficient between neurophysiologic and hand function measures at baseline

	Behavioral and Subjective Measures			
	AHA	Box and Blocks		COPM-Perf
**Hemisphere Assessed**		MA	LA	
Lesioned				
MT	-.71*	-.42	-.52	.31
Amplitude	.43	.46	.43	.43
CSP	NC	NC	NC	NC
# Map Sites	NC	NC	NC	NC
Non-Lesioned				
MT	-.60	-.50	-.52	.38
Amplitude	-.14	-.43	-.10	.53
CSP	-.09	.54	-.03	-.31
# Map Sites	-.20	.38	-.38	-.26

All assessments reflect an average of 4 pre-testing measures. Amplitudes and CSP duration correlations were conducted using the mean. *$p \leq 0.050$. AHA: Assisting Hand Assessment; COPM-Perf: Canadian Occupational Performance Measure – Performance Subtest; CSP: Cortical Silent Period; LA: Less-affected hand; MA: More-affected hand; MT: Motor Threshold; NC: Not calculated due to insufficient data.
